# Distribution of schistosomes and soil-transmitted helminth infections and their association with growth and nutritional status of School-aged children of the Matta health area in the West region of Cameroon

**DOI:** 10.1371/journal.pntd.0013606

**Published:** 2025-10-07

**Authors:** Estelle Mezajou Mewamba, Loic Edmond Tekeu Mengoue, Darelle Bethanie Motia, Rostand Joël Atiokeng Tatang, Arnol Auvaker Zebaze Tiofack, Gustave Simo

**Affiliations:** 1 Molecular Parasitology and Entomology Unit, Department of Biochemistry, Faculty of Science, University of Dschang, Dschang, Cameroon; 2 Centre for Research in Infectious Diseases (CRID), Yaoundé, Cameroon; 3 Research Unit of Biology and Applied Ecology, Department of Animal Biology, Faculty of Science, University of Dschang, Dschang, Cameroon; Central University of Tamil Nadu, INDIA

## Abstract

**Background:**

Updating and mapping the prevalence and infection intensities of schistosomiasis (SCH) and soil-transmitted helminth (STH) infections remain crucial to guide stakeholders in their decision of boosting or reducing control efforts. Although efforts have been made to achieve the elimination of these diseases, few considerations have been paid to their impact on the growth status of infected individuals. This study was designed to map the prevalence and infection intensities of schistosomiasis and soil-transmitted helminthiasis, and to assess their association with children’s growth status.

**Methodology:**

During a cross-sectional study, 870 urine and 764 stool samples were collected from school-aged children of four primary schools of Matta health area. Micro-hematuria and *Schistosoma haematobium* eggs were searched in urine samples, while *S. mansoni* and soil-transmitted helminths (STHs) eggs were investigated in stools. Schistosome and STHs species and their infection intensities were mapped, and associations between the infection status and growth parameters were assessed.

**Results:**

*Schistosoma haematobium* was the most prevalent and widespread parasite with an overall prevalence of 45.8%. *Schistosoma mansoni* was found in one village with a prevalence of 3.4%. The overall prevalence of STHs was 2.5% with *Ascaris lumbricoides* being the most abundant species (1.4%). Children from Mambonkor bord were the most infected and bearing heavy intensities of *S. haematobium* infections. Infected children were significantly more underweight than uninfected ones (*P = 0.03*). Micro-hematuria was significantly (*P *< 0.0001**) more detected in infected children compared to uninfected ones. Uninfected children were more stunted than infected children (*P =0.009*) while no significant difference was observed between boys and girls. Stunting (*P < 0.0001*) and wasting (*P < 0.0001*) were significantly more pronounced in children of five years.

**Conclusion:**

This study revealed that *S. haematobium* infections are widespread in villages of Matta health area, while *Schistosoma mansoni* infections were restricted only to Matta village. This study also showed a low circulation of STH infections in villages of Matta health area. The mapping revealed Mambonkor bord and Matta barrage as high transmission villages where control measures must be boosted to achieve schistosomiasis elimination. The underweight, stunting and wasting status observed in children of Matta health area were not associated with schistosome and STH infections.

## Author summary

Updating and mapping schistosome and soil-transmitted helminth (STH) infections and their infections intensities remain crucial to guide stakeholders in their decision of boosting or reducing control efforts. Although efforts have been made to achieve the elimination of these diseases, little attention is been paid to the impact of these infections on the growth status of children living in endemic areas. This study aimed to map the prevalence and infection intensities of these parasitic infections, and to assess their association with children’s growth status. After identifying *S. haematobium*, *S. mansoni* and soil-transmitted helminths (STHs), the prevalence and infection intensities of these parasites were mapped. Associations between these parasitic infections and growth parameters were performed. Results of this study showed a high and widespread of *S. haematobium* infections*.* They also showed a low prevalence of *S. mansoni* infections and its restriction in Matta village as well as a low circulation of STHs. The mapping revealed Mambonkor bord and Matta barrage as high transmission villages where control measures must be boosted. Schistosome and STH infections as well as their infection intensities were not associated with underweight, stunting and wasting that were observed in some children of the Matta health area.

## Introduction

Schistosomiasis (SCH) and Soil transmitted helminth (STH) infections are neglected tropical diseases that remain major public health problems for people living in impoverished vulnerable communities with limited access to safe water, sanitary facilities and inadequate health facilities [1]. These diseases cause severe morbidity and mortality in some of the poorest and most remote areas of sub-Saharan Africa. Significantly impacting the health, the economy and the development of affected countries, it is recognized that more than 40% of tropical diseases burden is due to SCH and STH [2].

Endemic in several countries especially in Africa where approximately 90% of cases are reported [3], 779 million people are at risk of contracting schistosomiasis with about 229 million people infected worldwide [4,5]. This disease particularly affects children and ranks second after malaria in terms of human suffering in the tropics and subtropics. In Cameroon, schistosomiasis is predominantly caused by two schistosomes species namely *Schistosoma haematobium* and *Schistosoma mansoni* which are responsible for urinary and intestinal schistosomiasis, respectively [6].

STH infections are a group of parasitic diseases that are transmitted to humans by fecally contaminated soil. They are caused by nematodes such as *Ascaris lumbricoides*, *Trichuris trichiuria*, and hookworms (*Necator americanus* and *Ancylostoma duodenale*). Commonly found in Asia, South America and Africa, more than 2 billion people are infected by at least one soil-transmitted helminth species worldwide [7]. Amongst the STH infections, those due to *A. lumbricoides*, *T. trichiura* and hookworms affect respectively 819, 464.6 and 438.9 million people worldwide [8].

Although several control strategies including improvement of water access, sanitation and hygiene initiatives (WASH), and the modification of people’s behaviour to reduce fecally contaminated environment have been developed for sustainable control in order to eliminate the transmission of schistosomiasis and STH infections [9,10], the control of these diseases has relied over the years mainly on the preventive chemotherapy (PC) by mass administrating anthelmintic drugs such as Praziquantel and Albendazole to school-aged children [11,12]. As this administration can be done once, twice a year or every two years depending on the disease prevalence in the endemic area [13], updating and mapping the disease prevalence is of paramount importance for the control programs. Although PC has enabled the considerable reduction in the prevalence of SCH and STH infections as well as their infection intensity and morbidity, it remains important to regularly update data on schistosome and STH infections in different endemic settings. Such data are critical for the monitoring of control programs and also for identifying areas showing low, moderate and high disease transmission and where the control measures must be modified by reducing or increasing the number of treatment rounds. Updating and mapping the disease prevalence and their infection intensities can provide critical information to support and guide stakeholders in their decision of boosting or reducing the control efforts that aim not only to achieve the diseases’ elimination, but also to improve human health by reducing the impacts associated with SCH and STH infections.

Although SCH and STH infections affect human health by inducing for instance hematuria and bladder cancer if not treated [14,15], their impacts have been overlooked in several communities. In Cameroon for instance, a persistent high rate of stunting and wasting has been reported in children below five years and also in school-aged children living in rural areas [16,17]. Though poverty, limited access to nutritious food, inadequate feeding, limited access to clean water and poor sanitation have been recognized as the main malnutrition drivers in children of low-and middle-income countries [18–21], the high burden of infectious diseases such as SCH and STH infections cannot be neglected [22,23]. Moreover, the effects of these infectious diseases on poor school performance, stunting, wasting and underweight status may vary according to the affected communities [18–21].

This cross-sectional study aimed to map the prevalence and infection intensities of schistosomiasis and soil-transmitted helminthiasis, and assess the association between these diseases and the growth status of children of Matta health area in the west region of Cameroon.

## Materials and methods

### Ethical statement

This study was approved by the National Ethics Committee for research on human health of the Ministry of Public Health of Cameroon with the reference number N°2023/08/1568/CE/CNERSH/SP. It was also approved by the review board of the Molecular Parasitology and Entomology Sub-unit of the Department of Biochemistry of the Faculty of Science of the University of Dschang. Approbations were also obtained from traditional and administrative authorities, school inspectors, directors and teachers. Parents or guardians of participating children approved their participation by signing the informed consent form on their behalf. In addition, children of 12–15 years signed assent forms while for younger children, only the consent form signed by their parents or guardians was considered. After a detailed explanation of objectives, procedures and potential risks and benefits of the study, each child and their parents or guardians were free to choose whether to participate or not to the study. Data from this study were anonymized during analyses. Results of parasitological and immunological tests were transmitted to parents or guardians and all children found with schistosome infections and/or soil transmitted helminth infections were treated respectively with praziquantel (40mg/Kg body weight) and albendazole (single oral dose of 400 mg) following WHO recommendations [24].

### Study area

**Fig 1:**
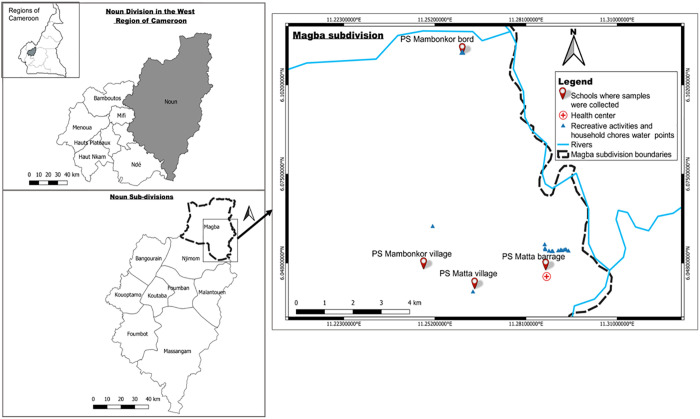
Map showing primary schools where children were sampled. PS: Public school, base layers of the map were obtained using a free online spatial data software (https://www.diva-gis.org/gdata).

This cross-sectional study was conducted in September 2023 in four villages of Matta health area of the Magba subdivision in the West region of Cameroon (Fig 1). This locality has a climate of equatorial type [25]. Its vegetation is made up of dense Savannah that is often muddy. Farming and fishing are the main inhabitants’ activities and also their main source of livelihood [26]. Matta health area is endemic for urinary schistosomiasis for more than ten years [6,25–27]. In this health area, mass drug administration of Praziquantel and anthelmintic drugs to school-aged children is the main control method used to fight against schistosomiasis and STH infections [28].

Although Matta health area has 16 villages, only four of them have four primary schools: the Public school (PS) of Matta barrage, the PS of Matta village, the PS of Mambonkor bord and the PS of Mambonkor village. The other villages are islands with difficult accessibility.

### Study design and population

This cross-sectional study involved school-aged children of five to 15 years attending the public school of Matta barrage, the public school of Matta village, the public school of Mambonkor bord and the public school of Mambonkor village. All school-aged children who signed the assent form and for whom a signed informed consent form was obtained from their parents or guardians were invited to participate in this study.

### Exclusion criteria

Participants who accepted to participate in the study and who were not able to provide either urine or stool samples were excluded from this study. Children who have received Praziquantel or other anthelmintic agents for the last two months were also excluded from this study.

### Sample collection

Each child who provided a signed assent form and had provided a signed informed consent form from their parents or guardians was invited to provide urine and stool samples in clean and well-labelled plastic containers. Approximately 50 mL of urine and five to seven grams of stool samples were collected from each child between 9:00 am and 1:00 pm. These samples were immediately transferred to the local health center where the urine filtration test (UF) and urine strip tests were performed on urine and the Kato-Katz test (KK) on stool samples.

### Detection of micro-hematuria in urine samples

The presence of micro-hematuria was assessed by inserting a urine strip in 50 mL of urine sample for 20 seconds as described by the manufacturer. Thereafter, the strip was removed and left at room temperature for 60 seconds before being read. When the strip color moved from yellow to green, the child was considered to have micro-hematuria in his urine.

### Detection of *S. haematobium* infections

*Schistosoma haematobium* eggs were identified in urine samples using the urine filtration test kit (Lot N°: M/310322, batch N°: 64720, Sterlitech Corporation, USA) as described by Peters et al. [29]. Each urine sample was vigorously shaken and thereafter, ten milliliters were filtered through a 12 μm polycarbonate filter provided by the manufacturer. The filter was removed and placed on a microscope slide and a drop of Lugol’s iodine solution was added to the slide before its microscopic examination [30]. For each positive slide, the number of eggs was counted and expressed as the number of eggs per ten milliliters of urine [30]. Samples with less than 50 eggs were considered as having light infection intensities while those with the number of eggs greater than 50 were considered as having high infection intensities.

### Detection of *Schistosoma mansoni* and soil-transmitted helminth infections

Schistosome and soil-transmitted helminth eggs were identified using the kato-katz technique as described by Katz et al. [31]. From each stool sample, a single KK thick smear slide was prepared with 41.7 mg of stool using Sterlitech kit (Lot: XGACAI). Briefly, a small quantity of stool was transferred onto a piece of scrap paper. Thereafter, a nylon mesh was pressed on the top of the fecal sample and a small plastic spatula was used to scrape the sieved material from the nylon screen. Subsequently, the well of the KK template was placed on a clean microscopic slide and completely filled with sieved fecal material. After removing the template without disturbing the calibrated fecal material, the slide was covered with a cellophane strip pre-soaked for 24 hours in glycerol-malachite green solution (a mixture of 100 mL of mineral water, 100 mL of glycerin and 1 mL of 3% green malachite solution). The stool was spread into a thick smear by inverting the microscope slide and pressing the stool sample against the cellophane on a smooth surface. The slides were microscopically observed after 24 hours at 10X magnification. Eggs of *S*. *mansoni* and those of different soil-transmitted helminth species were morphologically identified by examining each microscope slide. Samples were classified as having light, moderate or heavy *S. mansoni* infections when the number of eggs per gram of stool was respectively *<*100 EPG, 100–400 EPG and *>*400 EPG [24]. Each soil-transmitted helminth species was separately counted and its infection intensity determined as described by Montresor et al. [32]. For. *A. lumbricoides*, the infection intensities were considered as light, moderate and heavy when the number of eggs per gram of feces were respectively between 1–4999 EPG, 5000–49999 EPG and ≥50 000 EPG; for *T. trichiura*, the infection intensities were light for 1–999 EPG, moderate for 1000–9999 EPG and heavy for ≥ 10 000 EPG. For the hookworms, children were considered as carrying light, moderate and heavy infection intensities when the number of eggs per gram of stool were respectively between 1–1999 EPG, 2000–3999 EPG and ≥4000 EPG [32].

During sampling, the geographical coordinates of water points and those of four schools were recorded using a global positioning system (eTrex, Garmin International, Olathe, KS, USA).

### Mapping the schistosome and soil-transmitted helminth infections as well as their infection intensities

The Cameroon map containing the administrative divisions was downloaded using the link https://www.diva-gis.org/gdata (consulted, 11/04/2024). Thereafter, the geographical information layers of the road and hydrographic networks were projected onto the map using the geographical information system software (QGIS v.3.16.10 ’Hannover’; QGIS Development Team, https://qgis.org/downloads/QGIS-OSGeo4W-3.16.10-1-Setup-x86_64.exe). In addition to that, the geographical coordinates of schools and water points (where children perform risky activities such as swimming, bathing, fetching water, washing clothes or dishes) were inserted onto the map of endemic regions using the QGIS software. This software was subsequently used to map *S. haematobium*, *S*. *mansoni* and soil-transmitted helminth infections as well as their infection intensities.

### Collection of anthropometric data

From each child, the body weight was measured using a calibrated weighing scale while the height was measured using a stadiometer. The body mass index (BMI, kg/m^2^) was estimated as described by Nuttall [33]. Online software was used to assess stunting and wasting in each child by determining the values of height for age Z-score and BMI for age Z-score. This incorporates the Growth Reference Standard for children and adolescents from two to 20 years. The height for age Z-score (HAZ) was determined in boys and girls using “CDC height for age percentiles for boys calculator” (https://reference.medscape.com/calculator/653/cdc-height-for-age-percentiles-for-boys-2-20-years) and “CDC height for age percentiles for girls calculator” (https://reference.medscape.com/calculator/655/cdc-height-for-age-percentiles-for-girls-2-20-years#) online software. The BMI for age Z-score (BAZ) was also determined for boys and girls using the “body mass index percentiles for boys calculator” (https://www.msdmanuals.com/professional/multimedia/clinical-calculator/body-mass-index-bmi-percentiles-for-boys-2-to-20-years) and the “body mass index percentiles for girls calculator” (https://www.msdmanuals.com/professional/multimedia/clinical-calculator/cdc-weight-for-age-percentiles-for-girls-2-20-years) online software.

Children’s nutritional and growth status were assessed using anthropometric indicators. Children having height-for-age (HAZ) <− 2 SD and BMI for age (BAZ) <− 2 SD were considered as having stunting and wasting, respectively [34].

### Data analysis

Chi-square test was used to compare the prevalence of schistosome and STH infections between schools, ages and sexes, and also the proportion of stunting, BMI and wasting according to sex, age and infection status. Following the test of Kolmogorov Smirnov, the Mann-Whitney *U* test was used to compare the arithmetic means of infection intensities between age groups and sex as well as the mean BMI according to sex and infection status. The Kruskall Wallis H nonparametric one-way analysis of variance (ANOVA) test enabled the comparison of the means of infection intensities according to schools and also the BMI means according to age. The kappa values with 95% CIs were used to determine the concordance between the urine filtration test and urine strips. These values were interpreted as described by Landis et al [35]. The test was considered significant when the p-value was below 0.05.

The distances between schools and the nearest water points were determined using proximity analysis in QGIS software. The linear regression model was used to associate the distances of schools to the nearest water points with schistosome infection intensities. It was also used to associate the BMI, HAZ and BAZ with the infection intensities of schistosomes and STHs. For this association, age and sex were used as covariates.

## Results

### Characteristics of the study population

This study involved 1158 children from four primary schools in four villages of Matta health area (Table 1). Eleven (<1%) children were excluded because their parents or guardians did not provide a signed informed consent while 202 were unable to provide urine and/or stool samples during the sampling day (Table 1). The 945 (81.6%) children who provided urine and/or stool included 491 (52%) boys and 454 (48%) girls and their mean age was 9.2 ± 2.6 (95% CI 9.0–9.3) years: 9.3 ± 2.7 (95% CI 9.0–9.6) for boys and 9.1 ± 2.5 (95% CI 8.8–9.3) for girls. Among these 945 children, 870 (92.1%) provided urine and 764 (80.8%) stool samples. Of these children, 689 (72.9%) provided both urine and stool samples while 181 (19.2%) provided only urine and 75 (7.9%) only stool samples.

**Table 1:** Number of children enrolled per school.

Village	NCS	NCPS	NCWP	NR
**PS Matta barrage**	707	526	174	7
**PS Matta village**	233	215	16	2
**PS Mambokor village**	140	134	5	1
**PS Mambonkor bord**	78	70	7	1
**Total**	1158	945	202	11

NCS: Number of children enrolled in the study; NCPS: Number of children who provided urine and/or stool samples; NCWP: Number of children willing to participate but who were unable to provide samples; NR: Number of excluded children.

### Prevalence of *Schistosoma haematobium* infections according to schools, age groups and sex

From the 870 urine samples examined, 400 (45.8%) harboured *S. haematobium* eggs; giving thus a prevalence of urinary schistosomiasis of 45.8% (95%CI: 41.6-50.7). The highest prevalence of 66.1% was recorded in the public school of Mambonkor bord (95% CI: 47.4-89.7) and the lowest prevalence of 11.0% (95% CI: 6.8-16.9) in the public school of Matta village (Table 2, S1 Fig). Comparing the prevalence of urinary schistosomiasis, a significant difference (*χ2 = 123.2*, *p < 0*.*0001*, **df* = 3*) was observed between schools (Table 2).

**Table 2:** Prevalence of *S. haematobium* infections according to school, sex and age groups.

Factors	Number of urine sample analyzed	Number of children infected with *S. haematobium* (%)	95% CI^a^	Mean of egg per 10mL of urine ± SD	95%CI^b^
**Schools**					
PS Matta barrage	498	277 (55.6)	49.2-62.5	247.9 ± 1152.4	246.0-249.7
PS Matta village	190	21(11.0)	6.8-16.9	28.9 ± 45.6	26.6-31.2
PS Mambonkor bord	62	41 (66.1)	47.4-89.7	227.8 ± 410.8	223.2-232.4
PS Mambonkor village	120	61 (50.8)	38.9-65.3	192.2 ± 371.4	188.8-195.8
Total	870	400 (45.8)	41.6-50.7	225.8 ± 979.2	224.4-227.3
**P value**		**<0.0001** ^**a**^		**0.07** ^**b**^	
**χ** ^**2**^		**123.2**		**–**	
***H***		**–**		**6**	
**Sex**					
Boys	455	232(51)	44.6-57.9	229.8 ± 935.2	227.9-231.8
Girls	415	168 (40.5)	34.6-47.1	220.4 ± 1039.8	218.1-222.6
Total	870	400 (45.8)	41.6-50.7	225.8 ± 979.2	224.4-227.3
**P value**		**0.002** ^**a**^		**0.68** ^**c**^	
**χ** ^**2**^		**9.2**		**–**	
***U***		**–**		**19023**	
**By age**					
< 10	426	194 (45.5)	39.3-52.4	228.3 ± 974.9	226.2- 230.4
≥ 10	444	206 (46.4)	40.3-53.2	223.6 ± 985.7	221.5-225.6
Total	870	400 (45.8)	41.6-50.7	225.8 ± 979.2	224.4-227.3
**P value**		**0.8** ^**a**^		**0.34** ^**c**^	
**χ** ^**2**^		**0.03**		**–**	
***U***		**–**		**18880**	

NSA: Number of stool samples analyzed; EPG: egg per gram of stool; SD: standard deviation; χ2: chi-square test; H: Kruskal Wallis H test; U: Mann Whitney U test; a: P-value for chi-square test; b: P-value for Kruskal Wallis H test; c: P- value for Mann Whitney U test; 95% CIa: 95% confidence interval associated to prevalence; 95% CIb: 95% confidence interval associated to the mean of infection intensity.

*Schistosoma haematobium* eggs were detected in urine samples from 232 (51%: 232/455) boys compared with 168 from girls (40.5%:168/415). As such, the prevalence of *S. haematotium* infections was significantly (*χ2 = *9.2**, *p = 0.002*, **df* = 1*) higher in boys (51%) compared to girls (40.5%) (Table 2, S1 Fig).

Among the 426 children below ten years, 194 (45.5%: 194/426) had *S*. *haematobium* eggs in their urine, compared with children 10 and older where eggs were detected in 46.4% (206/444; Table 2). Comparing the prevalence of *S*. *haematobium* infections, no significant (*χ2 = 0.03*, *p = 0.8*, **df* = 1*) difference was observed between age groups (Table 2, S1 Fig).

### *Schistosoma haematobium* infection intensities according to schools, age groups and sex

Among the 400 children having *S*. *haematobium* infections, 252 (63%) and 148 (37%) had respectively light and heavy infection intensity. The highest infection intensity of 247.9 ± 1152.4 (95%CI: 246.0-249.7) eggs per ten milliliters of urine was recorded in children from the public school of Matta barrage and the lowest intensity of 28.9 ± 45.6 (95%CI: 26.6-31.2) in those from the public school of Matta village (Table 2). Comparing these infection intensities, no significant difference (*H = 6*, *p = 0*.07, **df* = 3)* was observed between schools (Table 2). The means of infection intensities were respectively 229.8 ± 935.2 (95% CI 227.9-231.8) and 220.4 ± 1039.8 (95% CI 218.1-222.6) eggs per ten milliliters of urine in boys and girls (Table 2). Comparing these means of infection intensities, no significant difference (*U = 19023*, *p = 0*.*68*, *df = 1*) was recorded between boys and girls (Table 2). Although the means of infection intensities were high in school-aged children below ten years (228.3 ± 974.9 eggs per ten milliliters of urine: 95%CI: 226.2-230.4) compared to those having at least ten years (223.6 ± 985.7 eggs per ten milliliters of urine: 95%CI: 221.5-225.6), no significant difference (*U = 18880*, *p = 0*.*34*, *df = 1*) was recorded between these age groups.

### Proportion of micro-hematuria and concordance between urine strip and urine filtration test

Of the 824 urine samples that were tested with urine strips, 354 (42.9%) had micro-hematuria. Among these 354 children, 279 (78.8%: 279/354) had *S. haematobium* infections while no infection was found in 75 children (21.2%: 75/354). Among children with micro-hematuria, a significant difference (*p < 0.0001*, *χ2 = *232.82**) was recorded between infected and uninfected ones (S2 Fig).

Concordant results between micro-hematuria and urine filtration tests were recorded for 648 (78.6%) samples: 279 (33.9%) and 369 (44.8%) samples were respectively positive and negative for both tests. Discordant results were obtained for 176 (21.4%) samples: 75 without *S. haematobium* eggs had micro-hematuria while micro-hematuria was not detected in 101 urine samples having *S. haematobium* eggs. Between the urinary filtration and urine strips tests, the concordance index expressed as the Kappa coefficient was 0.57 (*p < 0*.*0001*, 95% CI: 0.51-0.62); indicating a fair strength of agreement between the results of these two tests.

### Prevalence of *Schistosoma mansoni* infections according to schools, age groups and sex

From 764 children who provided stool samples, 26 harbored *S*. *mansoni* eggs; giving a prevalence of 3.4% (95% CI 2.2-5.0) for intestinal schistosomiasis. The highest prevalence of 13.6% (95% CI: 8.9-20) was recorded in children of the public school of Matta village while no *S*. *mansoni* egg was observed in those of the public schools of Matta barrage, Mambonkor bord and Mambonkor village. Comparing the prevalence of *S*. *mansoni* infections, a significant difference (*χ*2 = 80**.*7*, *p < 0*.*0001*, *df = 3*) was recorded between schools (Table 3, S1 Fig). No significant difference was recorded in the prevalence of intestinal schistosomiasis between boys and girls (χ*2 = 3*.8*4*, *p = 0*.*05*, *df = 1*), and also between age groups (χ*2 = 0.12*, *p = 0*.*72*, *df = 1*) (Table 3, S1 Fig).

**Table 3:** Prevalence of *S. mansoni* infections according to school, sex and age groups.

Factors	NSA	*S. mansoni* infections (%)	95% CI^a^	Mean EPG ± SD	95%CI^b^
**Schools**					
PS Matta barrage	400	0 (0)	–	–	–
PS Matta village	191	26(13.6)	8.9-20	126.5 ± 134.8	122.2-130.9
PS Mambonkor bord	49	0 (0)	–	–	–
PS Mambonkor village	124	0 (0)	–	–	–
Total	764	26 (3.4)	2.2-5.0	–	–
**P value**		**<0.0001** ^**a**^		**–**	
**χ** ^**2**^		**80.7**		**–**	
***H***		**–**		**–**	
**Sex**					
Male	394	8(2)	0.9-4.0	96 ± 82.1	89.3-103
Female	370	18 (4.9)	2.8-7.7	140 ± 152.7	134.6-145.6
Total	764	26 (3.4)	2.2-5.0	126.5 ± 134.8	122.2-130.9
**P value**		**0.05** ^**a**^		**0.53** ^**b**^	
**χ** ^**2**^		**3.84**		**–**	
***U***		**–**		**60.0**	
**Age group**					
< 10	393	12 (3)	1.6-5.3	154 ± 118.2	147- 161.2
≥ 10	371	14 (3.7)	2-6.3	102.9 ± 147.8	97.6-108.3
Total	764	26	2.2-5.0	126.5 ± 134.8	122.2-130.9
**P value**		**0.72** ^**a**^		**0.1** ^**b**^	
**χ** ^**2**^		**0.12**		**–**	
***U***		**–**		**52.0**	

NSA: Number of stool samples analyzed; EPG: egg per gram of stool; SD: standard deviation; χ2: chi-square test; H: Kruskal Wallis H test; U: Mann Whitney U test; a: P-value for chi-square test; b: P- value for Mann Whitney U test; 95% CIa: 95% confidence interval associated to prevalence; 95% CIb: 95% confidence interval associated to the mean of infection intensity.

### *Schistosoma mansoni* infection intensities according to schools, age groups and sex

Of the 26 children carrying *S*. *mansoni* eggs, 16 (61.5%), 9 (34.6%) and 1 (3.8%) had respectively light, moderate and heavy infection intensities. In children from the public school of Matta village, the mean number of eggs per gram of stool was 126.5 ± 134.8 EPG (Table 3). These means were 96 ± 82.1 EPG (95% CI 89.3-103) in boys, 140 ± 152.7 EPG (95% CI 134.6-145.6) in girls, 102.9 ± 147.8 EPG in children having at least ten years and 154 ± 118.2 EPG in those below ten years (Table 3). Comparing these means of infection intensities, no significant difference was recorded between boys and girls (*U = 60.0*, *p = 0*.*53*, *df = 1*), and also between age groups (*U = 52.0*, *p = 0*.*1*, *df = 1)* (Table 3).

### Prevalence of soil-transmitted helminth infections according to schools, age groups and sex

Of the 764 children who provided stool samples, 19 had eggs of at least one STHs species; giving an overall prevalence of 2.5% (95% CI 1.5-3.9). The prevalence of *A. lumbricoides* of 1.4% (95% CI: 0.7-2.6) was higher compared to 1.0% (95% CI: 0.5-2.1) recorded for hookworm (Table 4, S3 Fig). The highest prevalence of *A. lumbricoides* of 8.1 (95% CI: 3.9-14.8) was recorded in children of the Public school of Mambonkor village (Table 4, S3 Fig). Children below ten years (2.5%: 95% CI: 1.2-4.7) were more infected than those aged 10 and older (0.2%: 95%CI: 0.007-1.5) (Table 4, S3 Fig). Comparing the prevalence of *A. lumbricoides* infections, significant differences were recorded between schools (*χ*2 = **45.8; p* *< 0.0001; df* *= *3*) and age groups (*χ*2 = *5.45*, **p* *= *0.02*, **df* *= *1*) (Table 4, S3 Fig). No significant difference (*χ*2 = *0.51*, **p* *= *0.*48, **df* = *1**) was recorded between boys and girls for *A. lumbricoides* infections (Table 4, S3 Fig).

**Table 4:** Prevalence of soil-transmitted helminths infections according to school, sex and age.

Factors	NSA	*A. lumbricoides* (%)	95%CI	Mean EPG^a^ ± SD	95%CI	Hookworm (%)	95%CI	Mean EPG^b^ ± SD	95%CI
**Schools**									
PS Matta barrage	400	1(0.25)	0.006-1.4	408	369.4-449.6	6(1.5)	0.6-3.3	84 ± 73.9	76.8-91.7
PS Matta village	191	0(0)	–	–	–	0(0)	–	–	–
PS Mambonkor bord	49	0(0)	–	–	–	0(0)	–	–	–
PS Mambonkor village	124	10 (8.1)	3.9-14.8	7336 ± 7559.9	7283.8-7390.1	2(1.6)	0.9-8.3	36 ± 16.9	28.2- 45.3
Total	764	11(1.4)	0.7-2.6	6706.9 ± 7470.1	6658.6-6755.5	8(1.0)	0.5–2.1	72 ± 66.7	62.2-78.1
**P value**		**<0.0001** ^**a**^		**0.36** ^**b**^		**0.3** ^**a**^		**0.43** ^**b**^	
**χ** ^**2**^		**45.8**		**–**		**3.7**		**–**	
***H***		**–**		**1**				**3**	
**Sex**									
Male	394	4(1)	0.2-2.6	2142 ± 1992.9	2096.9-2187.8	4(1)	0.2-2.6	66 ± 40.9	58.3-74.5
Female	370	7(1.9)	0.7-3.9	9315.4 ± 8317.9	9244.1-9387.2	4(1.1)	0.3-2.8	78 ± 92.7	69.6-87.2
Total	764	11(1.4)	0.7-2.6	6706.9 ± 7470.1	6658.6- 6755.5	8(1.0)	0.5–2.1	72 ± 66.7	62.2-78.1
**P value**		**0.48** ^**a**^		**0.16** ^**b**^		**1** ^**a**^		**0.68** ^**b**^	
**χ** ^**2**^		**0.51**		**–**		**<0.0001**		**–**	
***U***		**–**		**6**		**–**		**6**	
**Age group**									
< 10	393	10(2.5)	1.2-4.7	7137.6 ± 77289	7085.3-7190.2	3(0.8)	0.2-2.2	32 ± 13.9	25.9-39.1
≥ 10	371	1(0.2)	0.007-1.5	2400	2304.9-2498	5(1.3)	0.4-3.1	96 ± 75.9	87.6-104.9
Total	764	11(1.4)	0.7-2.6	6706.9 ± 7470.1	6658.6- 6755.5	8(1.0)	0.5–2.1	72 ± 66.7	62.2-78.1
**P value**		**0.02** ^**a**^		**0.91** ^**b**^		**0.66** ^**a**^		**0.14** ^**b**^	
**χ** ^**2**^		**5.45**		**–**		**0.20**		**–**	
***U***		**–**		**4**		**–**		**2**	

SD: standard deviation; χ2: chi-square test; H: Kruskal Wallis H test; U: Mann Whitney U test; a: P-value for chi-square test; b: P- value for Mann Whitney U test.

The highest prevalence of hookworm of 1.6% (95% CI: 0.9-8.3) was recorded in children attending the Public school of Mambonkor village (Table 4, S3 Fig). Comparing the prevalence of hookworm, no significant difference was recorded between schools (*χ*2 = *3.7*, *p = 0.3*, **df* *= *3*), age groups (*χ*2 = 0.20, **p* *= *0.66*, **df* *= *1*) and also between boys and girls (*χ*2 *< 0.0001*, **p* *= *1*, **df* *= *1*) (Table 4, S3 Fig).

### Infection intensity of soil-transmitted helminths according to schools, age groups and sex

The mean number of eggs per gram of stool was 6706.9 ± 7470.1 EPG for *A. lumbricoides* and 72 ± 66.7 EPG for hookworm (Table 4). Comparing these means of infection intensities, no significant difference was observed between boys and girls for **A. lumbricoides (U* *= *6*, **p* *= *0.16*, *df = 1)* and hookworm **(U* *= *6*, **p* *= *0.68*, *df = 1)* and also between age groups for **A. lumbricoides (U* *= *4*, **p* *= *0.91*, *df = 1)* and hookworm *(U* = *2*, **p* *= *0.14*, **df* = 1)* (Table 4).

Six children of the public school of Matta village had co-infections of *S. haematobium* and *S. mansoni.* No other schistosome co-infection was found in children of other schools.

### Association between *S. haematobium* infections and children nutritional status

The mean BMI for the entire study population was 15.65 ± 2.3 kg/m^2^: 15.60 ± 2.25 (from 9.15 to 33.05 kg/m^2^) in boys and 15.69 ± 2.35 (from 10.55 to 26.93 kg/m^2)^ in girls (Table 5). No significant difference (*U = 93645*, *p = 0.84*, *df = 1*) was found in the means of BMI between boys and girls (Table 5). Children of all age groups except those of 15 years old were underweight (<18.5 Kg/m^2^) (Table 5). Significant difference (*p < 0.0001*, *H = 261.22*) was observed for the mean of BMI between age groups; children of five and six years being the most underweight (Table 5). The means of BMIs were 15.60 ± 2.24 and 15.69 ± 2.34 respectively in *S*. *haematobium* infected and uninfected children (S4A Fig). Between these two groups, no significant difference (*U = 92655.5*, *p = 0.72*, **df* = 1*) was recorded in the BMI mean, nor the BMI mean and infection intensities (*H = 2.15*, *p = 0.34*, **df* = 1*) (S4B Fig). Among the 400 and 470 *S*. *haematobium* infected and uninfected children, 371 (92.8%: 371/400) and 415 (88.3%: 415/470) were respectively underweight (BMI < 18.5 Kg/m^2^). Comparing these proportions of underweight children, a significant difference (*p = 0.03*, *χ*^*2 *^*= 6.41*, *df = 1*) was observed between *S. haematobium* infected and uninfected children (S5 Fig).

**Table 5:** Variations of anthropometric parameters according to sex, age and infection status.

Category	N	BMI ± SD	95%CI	Stunting (%)	95%CI	Wasting	95%CI
**Sex**							
Boys	455	15.60 ± 2.25	15.24-15.97	31(6.8)	4.6-9.7	73 (16)	12.58-20.17
Girls	415	15.69 ± 2.35	15.32-16.08	28 (6.7)	4.48-9.75	52 (12.5)	9.36-16.43
Total	870	15.65 ± 2.3	15.38-15.91	59 (6.8)	5.16-8.75	125 (14.4)	11.96-17.12
**P value**		**0.84** ^**a**^		**1**		**0.17** ^**b**^	
**χ** ^**2**^		**–**		**<0.0001** ^**b**^		**1.90**	
***U***		**93645**		**–**			
**Age**							
5	70	13.95 ± 2.87	13.08-14.85	16 (22.9)	13.06-37.12	30 (42.8)	28.92-61.18
6	60	13.75 ± 1.63	12.83-14.72	5 (8.3)	2.7-19.44	18 (30)	17.78-47.41
7	98	14.74 ± 1.59	13.98-15.51	3 (3.1)	0.6-8.95	14 (14.3)	7.81-23.97
8	123	14.96 ± 1.39	14.28-15.66	3 (2.4)	0.5-7.13	11 (8.9)	4.46-16.02
9	75	15.52 ± 1.91	14.64-16.44	5 (6.7)	2.17-15.58	8 (10.7)	4.61-21.02
10	154	15.99 ± 2.06	15.36-16.63	8 (5.2)	2.24-10.24	15 (9.7)	5.45-16.06
11	110	16.14 ± 1.73	15.40-16.90	3 (2.7)	0.56-7.97	12 (10.9)	5.64-19.06
12	101	17.30 ± 2.54	16.50-18.13	8 (7.9)	3.42-15.60	7 (6.9)	2.79-14.28
13	46	17.44 ± 1.89	16.25-18.68	3 (6.5)	1.35-19.06	4 (8.7)	2.37-22.26
14	25	16.97 ± 2.40	15.38-18.65	3 (12)	2.47-35.07	5 (20)	6.49-46.67
15	8	18.59 ± 2.62	15.64-21.73	2 (25)	3.03-90.31	1 (12.5)	0.32-69.65
Total	870	15.65 ± 2.30	15.38-15.91	59 (6.8)	5.16-8.75	125 (14.4)	11.96-17.12
**P value**		**<0.0001** ^**c**^		**<0.0001** ^**a**^		**<0.0001** ^**a**^	
**χ** ^**2**^		**–**		**43.62**		**72.03**	
***H***		**261.22**		**–**			
**Infection status**							
Infected	400	15.60 ± 2.24	15.22-15.99	17 (4.2)	2.48-6.80	53 (13.2)	9.93-17.33
Uninfected	470	15.69 ± 2.34	15.32-16.04	42 (8.9)	6.44-12.08	72 (15.3)	11.99-19.29
**Total**	**870**	**15.65 ± 2.3**	**15.38-15.91**	**59 (6.8)**	**5.16-8.75**	**125 (14.4)**	11.96-17.12
**P value**		**0.72** ^**a**^		**0.009** ^**a**^		**0.44** ^**a**^	
**χ** ^**2**^				**6.78**		**0.59**	

N: Number of children, SD: standard deviation; χ2: chi-square test; H: Kruskal Wallis H test; U: Mann Whitney U test; a: P- value for Mann Whitney U test; b: P-value for chi-square test; c: P- value for Kruskal Wallis H test; 95% CI: 95% confidence interval.

Although a strong association (*p < 0.0001*, *t = 16.37*) was recorded between the means of BMI and age, no association was recorded between the means of BMI and the intensities of *S. haematobium* infections (*p = 0.61*, *t = 0.51*), and also between the means of BMI and sex (*p = 0.24*, *t = 1.18*).

### Association between *S. haematobium* infections and children growth status

Among the 870 *S. haematobium* tested for *S. haematobium*, 59 (6.8%: 59/870) had stunting (HAZ **<* *-2SD). Children of 15 years had the highest proportion of stunting (Table 5). Comparing these proportions, significant differences were recorded between *S. haematobium* infected and uninfected children (*p = 0.009*, *χ*^*2 *^*= 6.78*), and also between age groups (**p* *< 0.0001**, *χ*^*2 *^*= *43.62**) (Table 5). No significant difference (*p = 1*, *χ*^*2 *^*< 0.0001*) was recorded between girls and boys (Table 5). Although a strong association (*p < 0.0001*, *t = -5.23*) was recorded between HAZ and age for the stunting, no association was observed between stunting and *S. haematobium* infection intensities (*p = 0.69*, *t = *-0.40**), and also between stunting for girls and boys (*p = 0.17*, *t = 1.37*).

The mean BAZ was -0.87 ± 1.75 and 14.4% (125/870) of children were reported with wasting. A high proportion (42.8%) of five years old children were reported with wasting (Table 5). Comparing the proportion of children with wasting (BAZ < -2SD), a significant difference was observed between age groups (**p* *< 0.0001**, *χ*^*2 *^*= *72.03**). No significant difference (*p = 0.17*, *χ*^*2 *^*= 1.90*) was observed between girls and boys, and also between *S. haematobium* infected and uninfected children (*p = 0.44*, *χ*^*2 *^*< 0.59*) (Table 5). Although strong associations were found (*p < 0.0001*, *t = 6.17*) between children having wasting (BAZ < -2SD) and age, and also between wasting and boys or girls (*p = 0.02*, *t = *2.22**), no association (*p = 0.97*, *t = 0.04*) was recorded between children having wasting and the intensities of *S. haematobium* infections.

### Association between *S. mansoni*, hookworm and *Ascaris lumbricoides* infection intensities with nutritional and growth status of children

No association was recorded between the HAZ with *S. mansoni* infection intensities (*p = 0.53*, *t = 0.62*), between BAZ and *S. mansoni* infection intensities (*p = 0.19*, *t = 1.32*), between HAZ (*p = 0.06*, *t = -1.87*) and *A. lumbricoides* infection intensities, between BAZ and *A. lumbricoides* infection intensities (*p = 0.23*, *t = 1.20*), between HAZ and hookworms infection intensities (*p = 0.41*, *t = -0.82)* and also between BAZ and hookworms infection intensities (*p = 0.76*, *t = 0.30*).

### Spatial distribution of schistosomes infections and their infection intensities

**Fig 2:**
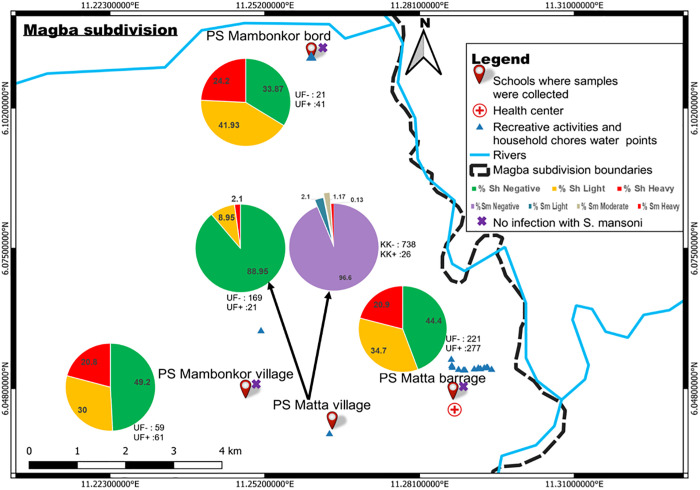
Distribution of *S. haematobium* and *S. mansoni* infections and their infection intensities according to villages of Matta health area. PS: Public school, Sh: *Schistosoma haematobium*, Sm: *Schistosoma mansoni*, KK + : Number of children carrying *S*. *mansoni* eggs in each village; KK-: Number of children without *S*. *mansoni* eggs in each village, UF + : Number of children carrying *S*. *haematobium* eggs in each village, UF-: Number of children without *S*. *haematobium* eggs in each village, base layers of the map were obtained using a free online spatial data software (https://www.diva-gis.org/gdata).

For this distribution, it was assumed that children attending each primary school were those living in that village. *Schistosoma haematobium* was the most frequently encountered parasite in the four villages (Fig 2). Children having heavy *S. haematobium* infection intensities lived mainly in Mambonkor bord village (Fig 2). The distance between this village and the nearest water point was the smallest (0.01 km) compared to 0.16 km for the Public school of Matta village, 0.64 km for the Public school of Matta barrage and 1.41 km for the public school of Mambonkor village. The highest proportion (24.2%) of children with heavy infection intensities was recorded in children from Mambonkor bord followed by those from Matta barrage (20.9%) characterized by its proximity to multiple recreative sites and household chores water points (Fig 2). Children with light infection intensities were predominant in Mambonkor bord, followed by those from Matta barrage, Mambonkor village and Matta village (Fig 2). *Schistosoma haematobium* uninfected children and very few infected ones bearing light and heavy infection intensities were predominant in Matta village. Children carrying *S. haematobium* infections were found in all villages while those with *S. mansoni* infections were recorded only in Matta village. Co-infections between urinary and intestinal schistosomiasis were recorded in children of Matta village (Fig 2).

### Spatial distribution of soil-transmitted helminth species

**Fig 3:**
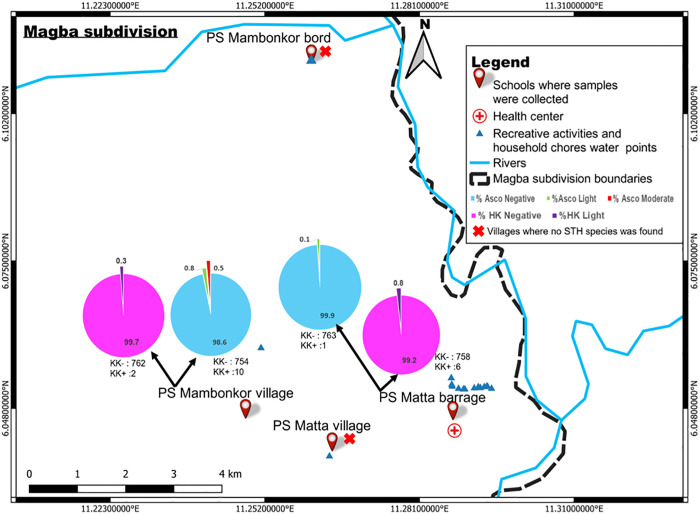
Distribution of soil-transmitted helminth species and their infection intensities according to villages of Matta health area. PS: Public school, Asco: *Ascaris lumbricoides,* HK: hookworm, KK + : Number of children carrying *Ascaris lumbricoides* eggs or hookworm eggs in each village; KK-: Number of children without *Ascaris lumbricoides* eggs or hookworm eggs in each village, base layers of the map were obtained using a free online spatial data software (https://www.diva-gis.org/gdata).

*Ascaris lumbricoides* and hookworm infections were found in children from Matta barrage and Mambonkor village. No heavy infection intensity of *A. lumbricoides* or hookworms was recorded in these children. Moderate infection intensities of *A. lumbricoides* were found in children from Mambonkor village while light infection intensities were found in those from Matta barrage and Mambonkor village. Children of these villages had light infection intensities of hookworms (Fig 3). No soil-transmitted helminth egg was found in children from Mambokor bord and Matta village (Fig 3).

### Relationship between the distances of houses of infected children andthe nearest water points

The correlation coefficients between the number of children bearing different *S. haematobium* infection intensities and the distances of schools to the nearest water points varied from -0.46 to -0.42 (Table 6). No significant correlation was observed between the distance of schools to the nearest water points and the number of children bearing light *S. haematobium* infection intensities (*p = 0.84*, *F = 0.05*), but also with the number of children bearing heavy infection intensities (*p = 0.77*, *F = 0.10*) (Table 6).

**Table 6:** Correlation coefficients between the mean distances of schools to the nearest water points and the infection intensities of *S*. *haematobium.*

School	DSNWP (km)	Light	Heavy
**PS Matta barrage**	0.62	173	104
**PS Matta village**	0.16	17	4
**PS Mambonkor bord**	0.01	26	15
**PS Mambonkor village**	1.41	36	25
**P**		0.84	0.77
**F**		0.05	0.10
**r** ^**2**^		-0.46	-0.42

PS: Public school; DSNWP: distance of school to the nearest water point; P: P-value; F: F- statistic from linear model

## Discussion

Results of this study show a widespread of urinary schistosomiasis in villages of Matta health area and high prevalence of *S. haematobium* infections of 45.8% compared to 16.6%, and 43% reported eleven and six years ago in this same locality [25,27]. This could be explained by the presence of the Mapé Dam and many temporary natural or artificial ponds for which the population depends for their water needs [25–27]. These results show a significantly high proportion of infected children with micro-hematuria in accordance with those reporting that hematuria is commonly found in children with *S. haematobium* infections [26,36]. Nevertheless, the presence of micro-hematuria in some children without *S. haematobium* eggs is in agreement with previous observations indicating the capacity of urine strips to yield some positive results not related to *S*. *haematobium* infections [37,38]. The kappa correlation index of 0.57 indicates a moderate strength of agreement between the urine filtration test and the presence of micro-hematuria in urine samples. This result indicates that detecting micro-hematuria could be considered as alternative approach to diagnose urinary schistosomiasis because hematuria is recognized as the main symptom of this disease [39,40].

The differences observed in the prevalence and infection intensities of *S*. *haematobium* between schools could be explained by the proximity of some schools to bio-ecological settings (rivers, dams, swimming points and sites for washing and/or fetching water) that are more favorable for schistosomiasis transmission. Results of this study showed a higher prevalence of urinary schistosomiasis in children of the public schools of Mambonkor bord and Matta barrage. This hypothesis is strengthened by the spatial distribution of *S. haematobium* infections and their infection intensities which shows that most infected children and those bearing heavy infection intensities were from Mambonkor bord and Matta barrage. These results on the prevalence and spatial distribution of *S. haematobium* and its infection intensities could be explained by the very close proximity of these villages to the Mapé dam which is an important source of *Bulinus truncatus*, the intermediate host of *S*. *haematobium* [25,26]. In addition to that, children of these villages are regularly exposed to schistosome infections and re-infections by frequently practicing risky activities in various favorable transmission biotopes that have been found in these villages; thus, increasing the risk of schistosome’s transmission [41,42]*.* In villages showing high prevalence of urinary schistosomiasis, control operations must be boosted by treating children and adults, but also by administering two rounds of praziquantel treatment. In addition to that, snail control, water sanitation and hygiene, dissemination of information, education and communication must be strengthened in these villages [43].

The absence of correlation between the infection intensities and the distance of school to the nearest water point indicates that the proximity of school to water point has no impact on the ability of children to harbor light or heavy *S*. *haematobium* infection intensities [43]. It also indicates that the risk for a child to carry different *S*. *haematobium* infection intensities does not depend on the distance between schools and the nearest water point, but from other factors such as the contact frequency with the infested water [25–27].

The significantly high prevalence of urinary schistosomiasis in boys is in line with those of some previous authors [25–27,44,45]. However, other studies reported a high prevalence of urinary schistosomiasis in girls [46,47]. The discrepancy between these results could be attributed to the behavioral difference of boys compared to girls in different epidemiological settings. In Matta health area, boys swim, bathe and fish frequently in water and hence, were more exposed to schistosome infections compared to girls.

Although the absence of significant differences in the prevalence of urinary and intestinal schistosomiasis according to sex and age groups is in agreement with previous data [48,49], these results are contrary to those of other studies reporting not only an increase prevalence of *S. mansoni* infections with age, but also a high prevalence of *S. haematobium* infections in children below ten years [26,27,48,50]. These discrepancies could be explained by the children’s behavior in different epidemiological settings [49]. In Matta health area, boys are more exposed to risky activities while children of five to nine years follow elders in their daily risky activities like swimming, washing clothes and fetching water. These children have the same level of exposure and henceforth, the same risk of contracting schistosome infections.

Results of the present study reporting for the first time *S. mansoni* infections in Matta health area could be explained by the fact that previous studies were particularly focused on urinary schistosomiasis and that intestinal schistosomiasis may have been overlooked. Moreover, the invasion of this schistosome species and/or its intermediate host in the area following the construction of the Mapé dam could not be excluded. This hypothesis is strengthened by data of Muzarabani et al. [51] reporting that constructing infrastructures like dams and dikes can create new snail breeding sites that can subsequently lead to the invasion of new trematodes and snail and/or the disappearance of existing snail species in settings within an endemic area. The pastoralists’ migration with their cattle during transhumance is another argument supporting the invasion of this schistosome species. Passing through different schistosomiasis endemic areas during the transhumance, pastoralists can acquire and ensure the transmission of different schistosome species including *S. mansoni* and/or *S. haematobium*.

The map showing co-infections of both *S. haematobium* and *S. mansoni* in children of Matta village indicates that Matta health area must be considered as endemic for urinary and intestinal schistosomiasis. Although no information was collected regarding where children with *S. mansoni* infections acquired these infections, the fact that this parasite was detected only in children of Matta village suggests its restricted transmission in this village. Malacological investigations targeting the *S. mansoni* intermediate host could help to identify potential transmission sites of intestinal schistosomiasis. The detection of *S. mansoni* infections in children of Matta village indicates not only co-transmission of urinary and intestinal schistosomiasis, but also the risk for *S. mansoni* to spread to neighboring villages.

Although the detection of *A. lumbricoides* and hookworm infections testified their presence in Matta health area, their low prevalence associated with moderate or low infection intensities indicates that this area is moving towards the elimination of soil-transmitted helminthiasis. The higher prevalence of *A. lumbricoides* infections compared to hookworms could be explained not only by the fact that its adult worms produce more eggs than other STHs species, but also by the ability of *A. lumbricoides* eggs to resist and persist for several months in adverse bio-ecological conditions and to subsequently ensure their transmission [52]. The variations observed in the prevalence of *A. lumbricoides* according to schools could be related to socioeconomic and bio-ecological differences between villages [53]. Our results showing no significant difference in the prevalence of *A. lumbricoides* infections according to sex corroborate those of previous studies [54–56]. These results could be explained by the fact that school-aged children have probably the same level of exposure to *A. lumbricoides* infections. Sharing the same poor socioeconomic and environmental conditions, boys and girls have the same probability of acquiring STH infections since these infections result from non-compliance of basic hygiene rules [57]. The significantly high prevalence of STHs in children below ten years could be explained by the fact that these children are usually involved in risky activities such as playing, walking barefoot and eating with dirty hands [58,59].

The low prevalence of hookworm infections can be explained by the transmission route, the sampled population and the short lifetime of infective larvae. As these larvae can stay in the soil for three to ten days, the probability for a treated child to be re-infected is low [60]. The absence of significant difference in the prevalence of hookworms between schools, sex and age groups could be explained by the fact that boys and girls of all ages share the same environment and most of them walk barefoot which exposes them to contaminated soil [54,56,61,62]. Our results showing not only the spatial distribution of STHs and their infection intensities, but also the co-infections of urinary and intestinal schistosomiasis in children of Matta village indicate the need of specific control measures to be implemented in this village.

Although no significant difference was recorded between the mean BMI in infected and uninfected children, results of this study showing underweight (BMI *< *18.5 Kg/m^2^) in boys and girls in all age groups (except children of 15 years) indicate that these children were malnourished with chronic energy deficiency. The low BMI (*<*18.5 Kg/m^2^) and the moderate levels of wasting and stunting recorded in infected children are in agreement with the undernutrition and stunting reported in schistosomiasis infected children of East Africa [21,63] and Brazil [64]. These results could be explained by the fact that worm infections reduce food intake and/or increase nutrient wastage via vomiting, diarrhea and blood loss [65]. The more pronounced stunting and wasting recorded in children of five years could be explained by the fact that they need a high amount of macro and micro-nutrients for rapid growth and brain development. These children are more subjected to malnutrition which has some impacts on their school performance [20,66,67]. The absence of association between schistosome infections as well as their infection intensities with the growth and nutritional parameters suggest that the stunting, the underweight and wasting recorded in children of Matta health area may not be absolutely due to schistosome and/or STH infections. Nevertheless, it is important to point out that in each individual, the parasitic infections followed by nutritional deficiency, immune deficiency and pathology depends broadly on occurring host microbiota and parasites interactions. The association between parasitic infection or the infection intensity with nutritional deficiency observed in individuals could be affected by the microbiota composition, the parasite species and the interactions between parasites and other microorganisms [68,69]. Indeed, nutritional deficiency occurs when the parasite and other microorganisms of microbiota compete for space and nutrients available in the gut [70]. Other factors such as the diet quality and the amount of food intake could play a role in the nutritional and growth status of these children. This hypothesis is strengthened by previous results showing an association between higher diet quality with a low risk of stunting in children of *S. mansoni* endemic areas [71]. As this study was performed only on school-aged children, no malacological investigation was performed in villages of Matta health area.

## Conclusion

This study revealed a high prevalence of urinary schistosomiasis and a very low prevalence of intestinal schistosomiasis and soil-transmitted helminthiasis in villages of the Matta health area of Cameroon. The detected parasites displayed morphological features consistent with those of the expected species and no unclassified parasite was observed during this study. This study also revealed co-infections of *S. mansoni* and *S. haematobium* in children of Matta village. The transmission of *S. haematobium* is probably high at Mambonkor bord while that of *A. lumbricoides* and hookworm is higher at Mambonkor village and Matta barrage. The restriction of *S. mansoni* infections in Matta village suggests further studies are required to understand the transmission of this parasite in this specific area. Results of this study showed that schistosome and STH infections as well as their infection intensities were not associated with being underweight, stunted or wasted. The stunting and wasting observed in children of Matta health area indicates the need to improve their diet.

## Supporting information

S1 Fig Prevalence of *S. haematobium* and *S. mansoni* according to schools (A), to sex(B) and age (C). (TIF)

S2 Fig Micro-hematuria in infected and uninfected children. (TIF)

S3 Fig Prevalence of *A. lumbricoides* and Hookworm according to schools (A), to sex(B) and age (C). (TIF)

S4 Fig Boxplots showing the distribution of Body mass index according to infection status (A) and infection intensities (B). (TIF)

S5 Fig Proportion of underweight children according to infection status. (TIF)

## References

[1] WHO. Ending the neglect to attain the Sustainable Development Goals–A road map for neglected tropical diseases 2021–2030. Geneva: World Health Organization. 2020.

[2] Schistosomiasis and soil-transmitted helminth infections--preliminary estimates of the number of children treated with albendazole or mebendazole. Wkly Epidemiol Rec. 2006;81(16):145–63. 16673507

[3] Hotez PJ, Kamath A. Neglected tropical diseases in sub-saharan Africa: review of their prevalence, distribution, and disease burden. PLoS Negl Trop Dis. 2009;3(8):e412. doi: 10.1371/journal.pntd.0000412 19707588 PMC2727001

[4] WHO. Schistosomiasis and soil-transmitted helminthiases: numbers of people Treated in 2018. Wkly Epidemiol Rec. 2018;94:601–12.

[5] Quansah R, Murad MH, Danso-Appiah T, Guuri C, Yakubu A, Cudjoe AB, et al. The effectiveness of praziquantel preventive chemotherapy on morbidity in schistosomiasis: a systematic review and meta-analysis. Cold Spring Harbor Laboratory. 2021. doi: 10.1101/2021.11.03.21265867

[6] Tchuem Tchuenté L-A, Kamwa Ngassam RI, Sumo L, Ngassam P, Dongmo Noumedem C, Nzu DDL, et al. Mapping of schistosomiasis and soil-transmitted helminthiasis in the regions of centre, East and West Cameroon. PLoS Negl Trop Dis. 2012;6(3):e1553. doi: 10.1371/journal.pntd.0001553 22413029 PMC3295801

[7] WHO. Soil-transmitted helminth infections. https://www.who.int/news-room/fact-sheets/detail/soil-transmitted-helminth-infections. 2024. 2024 July 13.

[8] Pullan RL, Smith JL, Jasrasaria R, Brooker SJ. Global numbers of infection and disease burden of soil transmitted helminth infections in 2010. Parasit Vectors. 2014;7:37. doi: 10.1186/1756-3305-7-37 24447578 PMC3905661

[9] Echazú A, Bonanno D, Juarez M, Cajal SP, Heredia V, Caropresi S, et al. Effect of Poor Access to Water and Sanitation As Risk Factors for Soil-Transmitted Helminth Infection: Selectiveness by the Infective Route. PLoS Negl Trop Dis. 2015;9(9):e0004111. doi: 10.1371/journal.pntd.0004111 26421865 PMC4589369

[10] Campbell SJ, Nery SV, McCarthy JS, Gray DJ, Soares Magalhães RJ, Clements ACA. A Critical Appraisal of Control Strategies for Soil-Transmitted Helminths. Trends Parasitol. 2016;32(2):97–107. doi: 10.1016/j.pt.2015.10.006 26795294

[11] WHO. Preventive chemotherapy in human helminthiasis: Coordinated use of anthelminthic drugs in control interventions: A manual for health professionals and programme managers. Geneva: World Health Organization. 2006.

[12] WHO. Helminth control in school-age children. A guide for managers of control programmes. Geneva: World Health Organization. 2011. http://apps.who.int/iris/bitstream/10665/44671/1/9789241548267_eng.pdf

[13] WHO. Schistosomiasis: progress report 2001–2011, strategic plan 2012–2020. World Health Organization. 2013.

[14] Gryseels B, Polman K, Clerinx J, Kestens L. Human schistosomiasis. Lancet. 2006;368(9541):1106–18. doi: 10.1016/S0140-6736(06)69440-3 16997665

[15] Efared B, Bako ABA, Idrissa B, Alhousseini D, Boureima HS, Sodé HC, et al. Urinary bladder Schistosoma haematobium-related squamous cell carcinoma: a report of two fatal cases and literature review. Trop Dis Travel Med Vaccines. 2022;8(1):3. doi: 10.1186/s40794-022-00161-x 35164874 PMC8845255

[16] Tabi ESB, Cumber SN, Juma KO, Ngoh EA, Akum EA, Eyong EM. A cross-sectional survey on the prevalence of anaemia and malnutrition in primary school children in the Tiko Health District, Cameroon. Pan Afr Med J. 2019;32:111. doi: 10.11604/pamj.2019.32.111.15728 31223401 PMC6560948

[17] Institut National de la Statistique I, ICF. Enquête démographique et de santé du Cameroun 2018. Yaoundé, Cameroun et Rockville, Maryland, USA: INS et ICF. 2020.

[18] Jukes MCH, Nokes CA, Alcock KJ, Lambo JK, Kihamia C, Ngorosho N, et al. Heavy schistosomiasis associated with poor short-term memory and slower reaction times in Tanzanian schoolchildren. Trop Med Int Health. 2002;7(2):104–17. doi: 10.1046/j.1365-3156.2002.00843.x 11841700

[19] de Gier B, Campos Ponce M, van de Bor M, Doak CM, Polman K. Helminth infections and micronutrients in school-age children: a systematic review and meta-analysis. Am J Clin Nutr. 2014;99(6):1499–509. doi: 10.3945/ajcn.113.069955 24740209

[20] Kasambala M, Mduluza T, Vengesai A, Mduluza-Jokonya T, Jokonya L, Midzi H, et al. Effect of Schistosoma haematobium infection on the cognitive functions of preschool age children and benefits of treatment from an endemic area in Zimbabwe. BMC Infect Dis. 2022;22(1):809. doi: 10.1186/s12879-022-07784-7 36316647 PMC9620666

[21] Mulindwa J, Namulondo J, Kitibwa A, Nassuuna J, Nyangiri OA, Kimuda MP, et al. High prevalence of Schistosoma mansoni infection and stunting among school age children in communities along the Albert-Nile, Northern Uganda: A cross sectional study. PLoS Negl Trop Dis. 2022;16(7):e0010570. doi: 10.1371/journal.pntd.0010570 35895705 PMC9359559

[22] Tanang TP. Facteurs explicatifs de la malnutrition des enfants de moins De cinq ans au Cameroun. 2009.

[23] Dama U, Tchoffo D, Onana Akoa FA, Abanda JN, Dzeuta MF, Asobochia AT, et al. Prévalence de la malnutrition chez les enfants de moins de 5 ans dans les départements du Mayo-Tsanaga et du Logone et Chari, Extrême-Nord, Cameroun. PAMJ-CM. 2024;14. doi: 10.11604/pamj-cm.2024.14.3.41534

[24] WHO Expert Committee. Prevention and control of schistosomiasis and soil-transmitted helminthiasis. World Health Organ Tech Rep Ser. 2002;912:i–vi, 1–57, back cover. 12592987

[25] Mewabo AP, Moyou RS, Kouemeni LE, Ngogang JY, Kaptue L, Tambo E. Assessing the prevalence of urogenital schistosomaisis and transmission risk factors amongst school-aged children around Mapé dam ecological suburbs in Malantouen district, Cameroon. Infect Dis Poverty. 2017;6(1):40. doi: 10.1186/s40249-017-0257-7 28260525 PMC5338087

[26] Njunda AL, Ndzi EN, Assob JCN, Kamga H-LF, Kwenti ET. Prevalence and factors associated with urogenital schistosomiasis among primary school children in barrage, Magba sub-division of Cameroon. BMC Public Health. 2017;17(1):618. doi: 10.1186/s12889-017-4539-6 28673343 PMC5496429

[27] Gilbert Gautier Bong B, Véronique M, Ghislaine Haverie Ateba M, Christian Taheu N, Philippe Salomon N. Urinary schistosomiasis prevalence and risk factors among school children at matta-barrage in the Tikar Plain of Magba, West Region, Cameroon: A situational analysis in rural area. World J Adv Res Rev. 2022;14(3):532–40. doi: 10.30574/wjarr.2022.14.3.0596

[28] Program Nationale de la Lutte Contre la Schistosomiase et les Helminths du Cameroun (PNLSH). Plan strategique 2005–2010. Cameroon: MINSANTE. 2005. https://pnlshi.org/publications/report

[29] Peters PA, Mahmoud AA, Warren KS, Ouma JH, Siongok TK. Field studies of a rapid, accurate means of quantifying Schistosoma haematobium eggs in urine samples. Bull World Health Organ. 1976;54(2):159–62. 1088097 PMC2366435

[30] Mewamba EM, Noyes H, Tiofack AAZ, Kamga RMN, Kamdem CN, Mengoue LET, et al. Association between polymorphisms of IL4, IL13, IL10, STAT6 and IFNG genes, cytokines and immunoglobulin E levels with high burden of Schistosoma mansoni in children from schistosomiasis endemic areas of Cameroon. Infect Genet Evol. 2023;111:105416. doi: 10.1016/j.meegid.2023.105416 36889485 PMC10167540

[31] Katz N, Chaves A, Pellegrino J. A simple device for quantitative stool thick-smear technique in Schistosomiasis mansoni. Rev Inst Med Trop Sao Paulo. 1972;14(6):397–400. 4675644

[32] Montresor A, Crompton DWT, Hall A, Bundy DAP, Savioli L. Guidelines for the evaluation of soil-transmitted helminthiasis and schistosomiasis at community level: a guide for managers of control programmes. World Health Organization. 1998. https://iris.who.int/handle/10665/63821

[33] Nuttall FQ. Body Mass Index: Obesity, BMI, and Health: A Critical Review. Nutr Today. 2015;50(3):117–28. doi: 10.1097/NT.0000000000000092 27340299 PMC4890841

[34] WHO. Software for assessing growth of the world’s children and adolescents. 2009. https://www.who.int/tools/growth-reference-data-for-5to19-years

[35] Landis JR, Koch GG. An application of hierarchical kappa-type statistics in the assessment of majority agreement among multiple observers. Biometrics. 1977;33(2):363–74. doi: 10.2307/2529786 884196

[36] Saotoing P, Vroumsia T, Am N, Tchuenguem FN, Messi J. Epidemiological survey of schistosomiasis due to Schistosoma haematobium in some primary schools on the town of Maroua, far north region Cameroon. Trop Med Int Health. 2011;6(2):19–24.

[37] King CH, Bertsch D. Meta-analysis of urine heme dipstick diagnosis of Schistosoma haematobium infection, including low-prevalence and previously-treated populations. PLoS Negl Trop Dis. 2013;7(9):e2431. doi: 10.1371/journal.pntd.0002431 24069486 PMC3772022

[38] Deribew K, Yewhalaw D, Erko B, Mekonnen Z. Urogenital schistosomiasis prevalence and diagnostic performance of urine filtration and urinalysis reagent strip in schoolchildren, Ethiopia. PLoS One. 2022;17(7):e0271569. doi: 10.1371/journal.pone.0271569 35877771 PMC9312429

[39] WHO. Schistosomiasis. https://www.who.int/news-room/fact-sheets/detail/schistosomiasis. 2024. 2024 July 15.

[40] Barsoum RS. Urinary schistosomiasis: review. J Adv Res. 2013;4(5):453–9. doi: 10.1016/j.jare.2012.08.004 25685452 PMC4293885

[41] Lamberti O, Kabatereine NB, Tukahebwa EM, Chami GF. Schistosoma mansoni infection risk for school-aged children clusters within households and is modified by distance to freshwater bodies. PLoS One. 2021;16(11):e0258915. doi: 10.1371/journal.pone.0258915 34735487 PMC8568121

[42] Arnold BF, Kanyi H, Njenga SM, Rawago FO, Priest JW, Secor WE, et al. Fine-scale heterogeneity in Schistosoma mansoni force of infection measured through antibody response. Proc Natl Acad Sci U S A. 2020;117(37):23174–81. doi: 10.1073/pnas.2008951117 32868437 PMC7502727

[43] Mewamba EM, Tiofack AAZ, Kamdem CN, Tchounkeu EY, Tatang RJA, Mengoue LET, et al. Fine-scale mapping of Schistosoma mansoni infections and infection intensities in sub-districts of Makenene in the Centre region of Cameroon. PLoS Negl Trop Dis. 2022;16(10):e0010852. doi: 10.1371/journal.pntd.0010852 36227962 PMC9595529

[44] Abdulkareem BO, Habeeb KO, Kazeem A, Adam AO, Samuel UU. Urogenital Schistosomiasis among Schoolchildren and the Associated Risk Factors in Selected Rural Communities of Kwara State, Nigeria. J Trop Med. 2018;2018:6913918. doi: 10.1155/2018/6913918 29853921 PMC5954937

[45] Opara KN, Akomalafe RT, Udoidung NI, Afia UU, Yaro CA, Bassey BE. Urogenital Schistosomiasis among Primary School Children in Rural Communities in Obudu, Southern Nigeria. Int J MCH AIDS. 2021;10(1):70–80. doi: 10.21106/ijma.407 33614224 PMC7873395

[46] Ndamukong KJ, Ayuk MA, Dinga JS, Akenji TN, Ndiforchu VA, Titanji VP. Infection pattern of Schistosoma haematobium in primary school children of the Kumba Health District, South-West Cameroon. Afr J Health Sci. 2000;7(3–4):98–102. 17650033

[47] Ntonifor HN, Green AE, Bopda MOS, Tabot JT. Epidemiology of urogenital schistosomiasis and soil transmitted helminthiasis in a recently established focus behind Mount Cameroon. Int J Curr Microbiol App Sci. 2015;4(3):1056–66.

[48] Angora EK, Boissier J, Menan H, Rey O, Tuo K, Touré AO, et al. Prevalence and Risk Factors for Schistosomiasis among Schoolchildren in two Settings of Côte d’Ivoire. Trop Med Infect Dis. 2019;4(3):110. doi: 10.3390/tropicalmed4030110 31340504 PMC6789509

[49] Mewamba EM, Tiofack AAZ, Kamdem CN, Ngassam RIK, Mbagnia MCT, Nyangiri O, et al. Field assessment in Cameroon of a reader of POC-CCA lateral flow strips for the quantification of Schistosoma mansoni circulating cathodic antigen in urine. PLoS Negl Trop Dis. 2021;15(7):e0009569. doi: 10.1371/journal.pntd.0009569 34260610 PMC8312929

[50] Sady H, Al-Mekhlafi HM, Mahdy MAK, Lim YAL, Mahmud R, Surin J. Prevalence and associated factors of Schistosomiasis among children in Yemen: implications for an effective control programme. PLoS Negl Trop Dis. 2013;7(8):e2377. doi: 10.1371/journal.pntd.0002377 23991235 PMC3749985

[51] Muzarabani KC, Carolus H, Schols R, Hammoud C, Barson M, Huyse T. An update on snail and trematode communities in the Sanyati Basin of Lake Kariba: New snail and trematode species but no human schistosomes. Parasitol Int. 2024;99:102830. doi: 10.1016/j.parint.2023.102830 38016629

[52] Jia T-W, Melville S, Utzinger J, King CH, Zhou X-N. Soil-transmitted helminth reinfection after drug treatment: a systematic review and meta-analysis. PLoS Negl Trop Dis. 2012;6(5):e1621. doi: 10.1371/journal.pntd.0001621 22590656 PMC3348161

[53] Kamdem CN, Tiofack AAZ, Mewamba EM, Tchounkeu EY, Tatang JRA, Mengoue ELT, et al. Fine mapping of Ascaris lumbricoides, Trichuris trichiura and hookworm infections in sub-districts of Makenene in Centre Region of Cameroun. Sci Rep. 2022;12(1):13935. doi: 10.1038/s41598-022-18285-7 35978014 PMC9385646

[54] Dankoni EN, Tchuem Tchuenté LA. Epidemiology of schistosomiasis and soil-transmitted helminthiasis in the sub-division of Kékem (West-Cameroon). IJIAS. 2014;8(4):1782–90.

[55] Ngonjo T, Okoyo C, Andove J, Simiyu E, Lelo AE, Kabiru E, et al. Current Status of Soil-Transmitted Helminths among School Children in Kakamega County, Western Kenya. J Parasitol Res. 2016;2016:7680124. doi: 10.1155/2016/7680124 27525108 PMC4971323

[56] Okoyo C, Campbell SJ, Williams K, Simiyu E, Owaga C, Mwandawiro C. Prevalence, intensity and associated risk factors of soil-transmitted helminth and schistosome infections in Kenya: Impact assessment after five rounds of mass drug administration in Kenya. PLoS Negl Trop Dis. 2020;14(10):e0008604. doi: 10.1371/journal.pntd.0008604 33027264 PMC7540847

[57] Joël ATR, Jeannette Y, Arlette NT, Vanessa N, Mbida M. Soil-Transmitted Helminths: Prevalence and Intensity of Some Soil Transmitted Nematodes among Pupils in Selected Primary Schools in Penka-Michel Sub-division, West-Cameroon. IJTDH. 2020;:11–22. doi: 10.9734/ijtdh/2020/v41i630282

[58] Nute AW, Endeshaw T, Stewart AEP, Sata E, Bayissasse B, Zerihun M, et al. Prevalence of soil-transmitted helminths and Schistosoma mansoni among a population-based sample of school-age children in Amhara region, Ethiopia. Parasit Vectors. 2018;11(1):431. doi: 10.1186/s13071-018-3008-0 30041691 PMC6056938

[59] Bishop HG, Azeez Z, Momoh SJ, Abdullahi B, Ujah AO. Risk factors and effects of hookworm infections on anthropometric indices of school children in Samaru, Zaria, Nigeria. Science World Journal. 2022;17(2):291–4.

[60] Brooker S, Clements ACA, Bundy DAP. Global epidemiology, ecology and control of soil-transmitted helminth infections. Adv Parasitol. 2006;62:221–61. doi: 10.1016/S0065-308X(05)62007-6 16647972 PMC1976253

[61] Jiraanankul V, Aphijirawat W, Mungthin M, Khositnithikul R, Rangsin R, Traub RJ, et al. Incidence and risk factors of hookworm infection in a rural community of central Thailand. Am J Trop Med Hyg. 2011;84(4):594–8. doi: 10.4269/ajtmh.2011.10-0189 21460016 PMC3062455

[62] Joël ATR, Jeannette Y, Arlette NT, Vanessa N, Mbida M. Soil-Transmitted Helminths: Prevalence and Intensity of Some Soil Transmitted Nematodes among Pupils in Selected Primary Schools in Penka-Michel Sub-division, West-Cameroon. IJTDH. 2020;:11–22. doi: 10.9734/ijtdh/2020/v41i630282

[63] Corbett EL, Butterworth AE, Fulford AJ, Ouma JH, Sturrock RF. Nutritional status of children with schistosomiasis mansoni in two different areas of Machakos District, Kenya. Trans R Soc Trop Med Hyg. 1992;86(3):266–73. doi: 10.1016/0035-9203(92)90305-v 1412650

[64] Assis AMO, Prado MS, Barreto ML, Reis MG, Conceição Pinheiro SM, Parraga IM, et al. Childhood stunting in Northeast Brazil: the role of Schistosoma mansoni infection and inadequate dietary intake. Eur J Clin Nutr. 2004;58(7):1022–9. doi: 10.1038/sj.ejcn.1601926 15220944

[65] Lwanga F, Kirunda BE, Orach CG. Intestinal helminth infections and nutritional status of children attending primary schools in Wakiso District, Central Uganda. Int J Environ Res Public Health. 2012;9(8):2910–21. doi: 10.3390/ijerph9082910 23066405 PMC3447595

[66] Clark H, Coll-Seck AM, Banerjee A, Peterson S, Dalglish SL, Ameratunga S, et al. A future for the world’s children? A WHO-UNICEF-Lancet Commission. Lancet. 2020;395(10224):605–58. doi: 10.1016/S0140-6736(19)32540-1 32085821

[67] Pomati M, Nandy S. Assessing Progress towards SDG2: Trends and Patterns of Multiple Malnutrition in Young Children under 5 in West and Central Africa. Child Ind Res. 2019;13(5):1847–73. doi: 10.1007/s12187-019-09671-1

[68] Mukherjee S, Joardar N, Sengupta S, Sinha Babu SP. Gut microbes as future therapeutics in treating inflammatory and infectious diseases: Lessons from recent findings. J Nutr Biochem. 2018;61:111–28. doi: 10.1016/j.jnutbio.2018.07.010 30196243 PMC7126101

[69] Chakraborty P, Aravindhan V, Mukherjee S. Helminth-derived biomacromolecules as therapeutic agents for treating inflammatory and infectious diseases: What lessons do we get from recent findings?. Int J Biol Macromol. 2023;241:124649. doi: 10.1016/j.ijbiomac.2023.124649 37119907

[70] van den Elsen LW, Poyntz HC, Weyrich LS, Young W, Forbes-Blom EE. Embracing the gut microbiota: the new frontier for inflammatory and infectious diseases. Clin Transl Immunology. 2017;6(1):e125. doi: 10.1038/cti.2016.91 28197336 PMC5292562

[71] Kishino M, Hida A, Chadeka EA, Inoue M, Osada-Oka M, Matsumoto S, et al. Association between diet quality and risk of stunting among school-aged children in Schistosoma mansoni endemic area of western Kenya: a cross-sectional study. Trop Med Health. 2024;52(1):12. doi: 10.1186/s41182-023-00566-0 38233936 PMC10792916

