# Supplementary figures and images for: Distribution of schistosomes and soil-transmitted helminth infections and their association with growth and nutritional status of School-aged children of the Matta health area in the West region of Cameroon
Source: PLoS Negl Trop Dis. 2025 Oct 7;19(10):e0013606. doi: 10.1371/journal.pntd.0013606 (PMC12543179; doi:10.1371/journal.pntd.0013606)

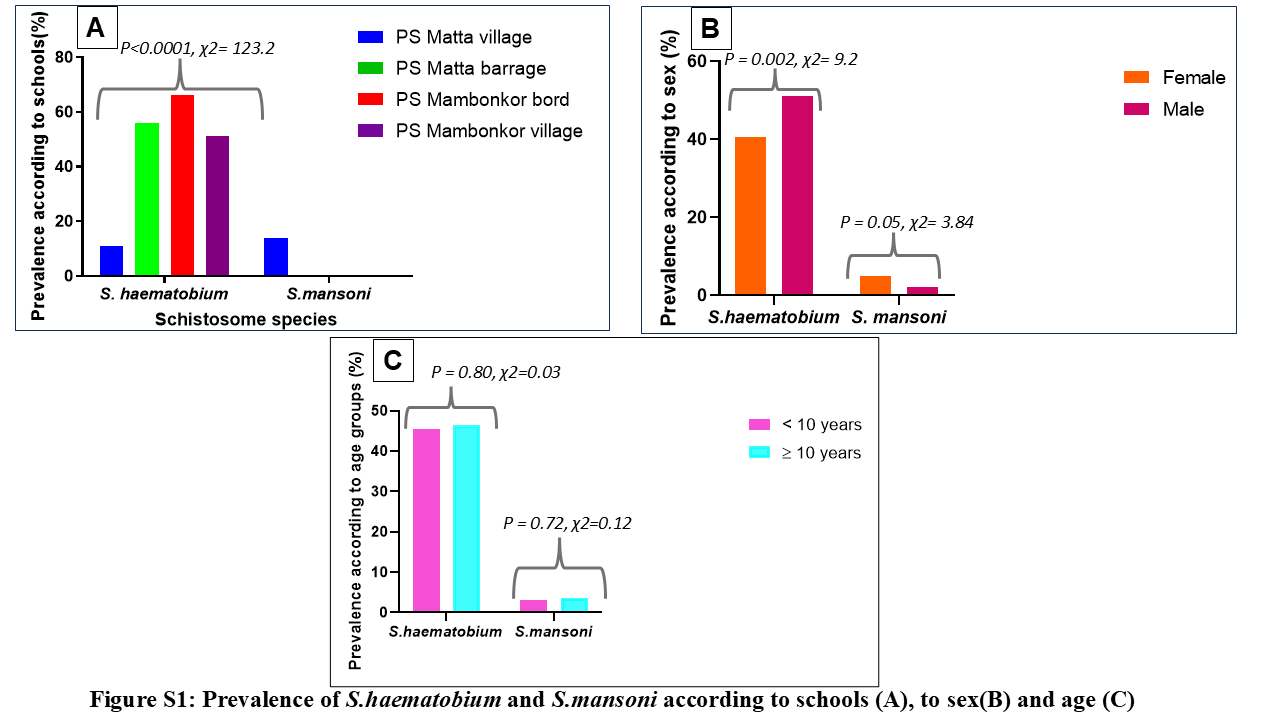

Supplement: S1 Fig — (TIF) [file pntd.0013606.s001.tif]

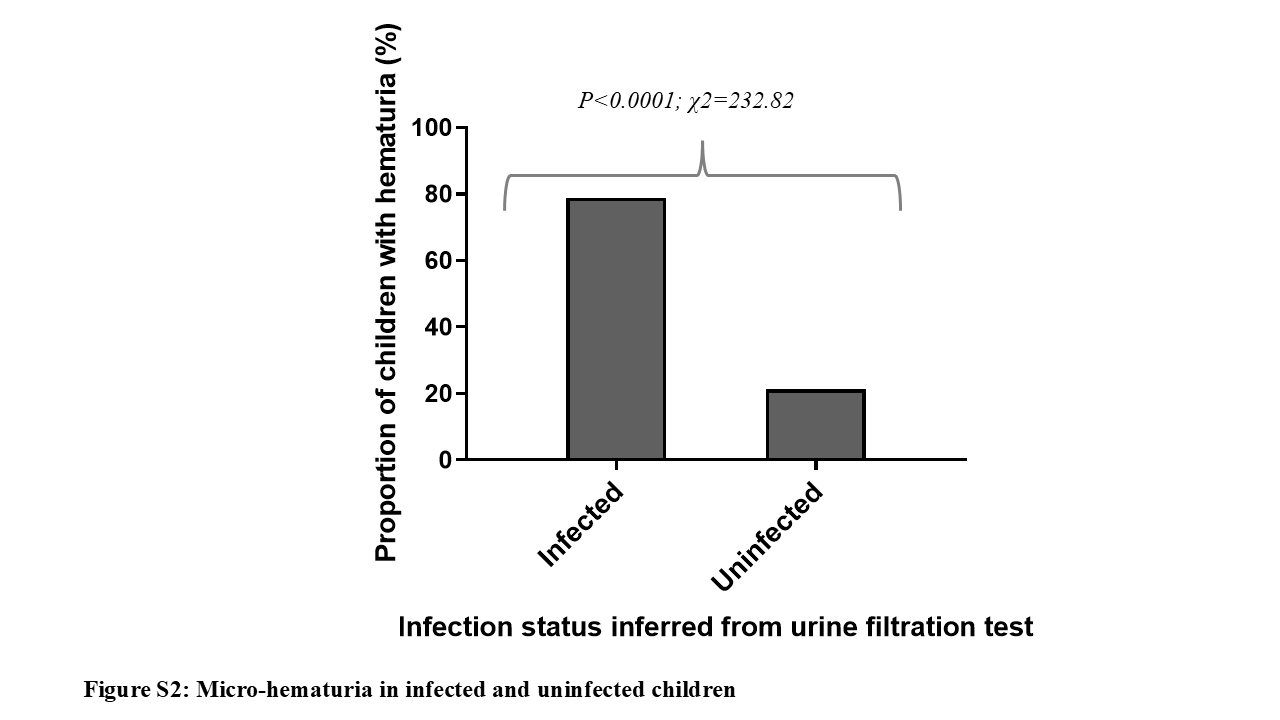

Supplement: S2 Fig — (TIF) [file pntd.0013606.s002.tif]

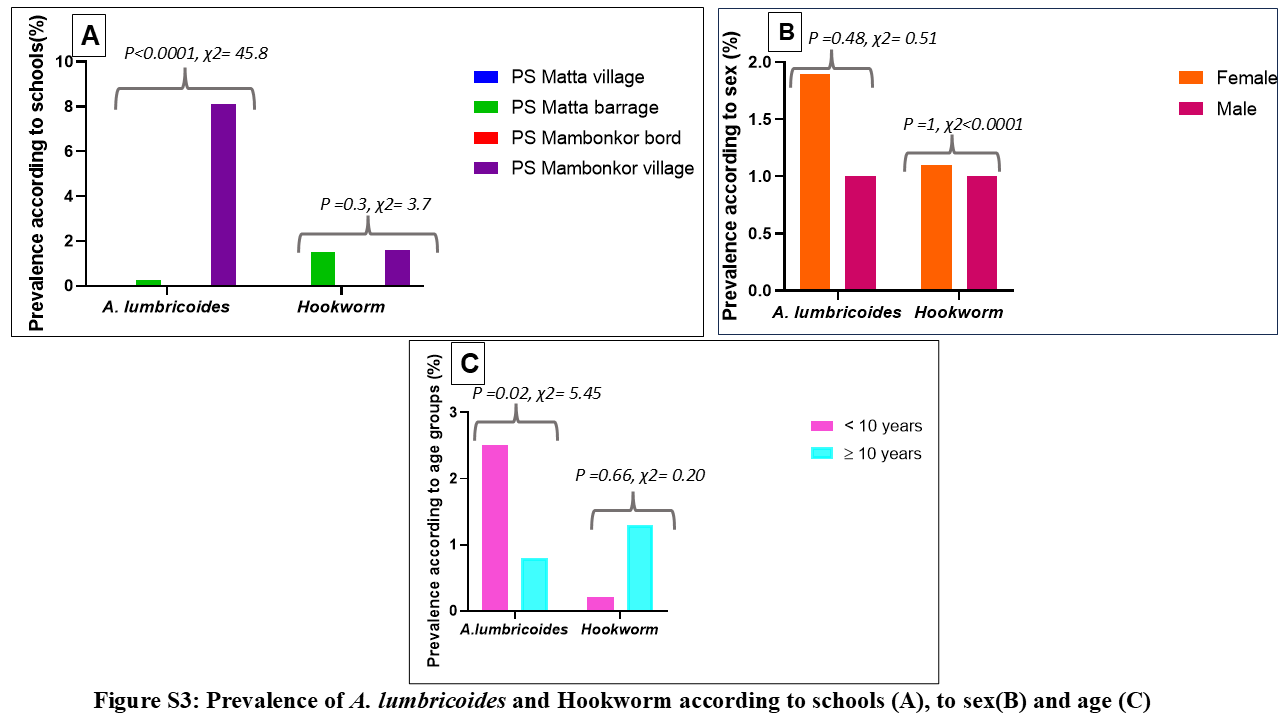

Supplement: S3 Fig — (TIF) [file pntd.0013606.s003.tif]

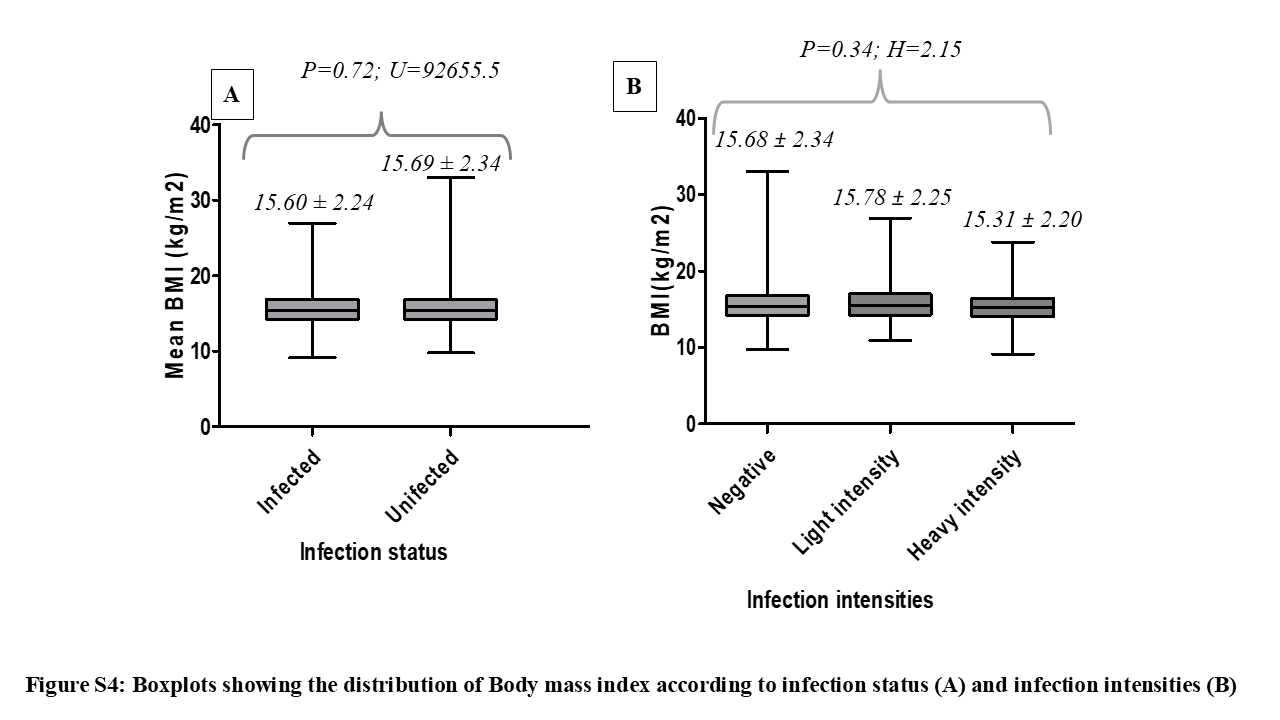

Supplement: S4 Fig — (TIF) [file pntd.0013606.s004.tif]

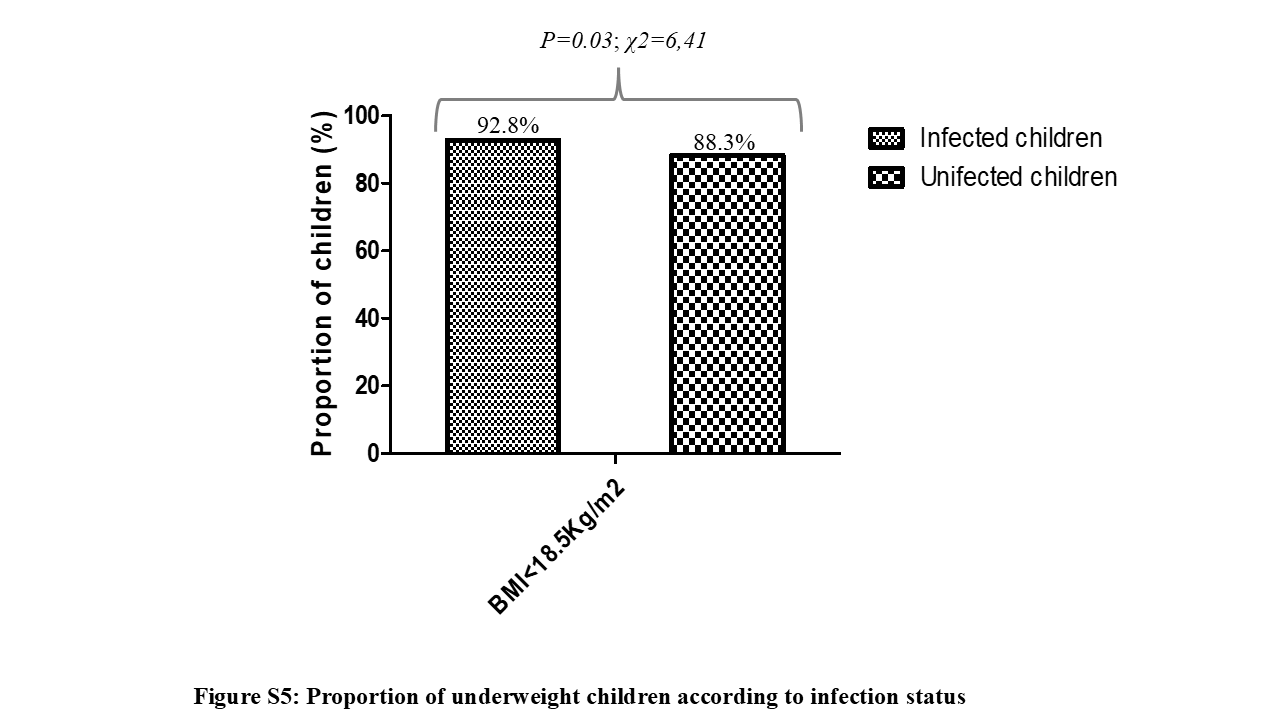

Supplement: S5 Fig — (TIF) [file pntd.0013606.s005.tif]
